# Urinary Proteomic Signatures in Sickle Cell Disease: Search for Biomarkers Associated With Underlying Molecular Pathways

**DOI:** 10.1155/ah/8684436

**Published:** 2026-09-15

**Authors:** Vera Hoving, Hans Wessels, Jolein Gloerich, Miranda van Berkel, Albertine E. Donker, Dorine W. Swinkels, Saskia E. M. Schols

**Affiliations:** ^1^ Department of Hematology, Radboud University Medical Center, Nijmegen 6525 GA 10, the Netherlands, radboudumc.nl; ^2^ Radboudumc Iron Expertise Center, Nijmegen 6525 GA 10, the Netherlands; ^3^ Department of Human Genetics, Translational Metabolic Laboratory, Radboud University Medical Center, Nijmegen 6525 GA 10, the Netherlands, radboudumc.nl; ^4^ Department of Laboratory Medicine, Radboud Laboratory for Diagnostics, Radboud University Medical Center, Nijmegen 6525 GA 10, the Netherlands, radboudumc.nl; ^5^ Department of Pediatrics, Maxima Medical Center, Veldhoven 5504 DB 4600, the Netherlands; ^6^ Sanquin Blood Bank, Amsterdam 1066 CX 125, the Netherlands

## Abstract

Identifying urinary biomarkers of sickle cell disease (SCD) may provide insights into disease mechanisms and support improved disease monitoring. In this pilot study, we analyzed the urinary proteome of 32 SCD patients (children and adults, steady state, and various genotypes) and 19 healthy controls using mass spectrometry‐based proteomics. A total of 2263 proteins were detected, of which 978 were consistently quantified across groups. Ninety proteins showed differential abundance between SCD patients and controls, with 26 upregulated and 64 downregulated in SCD. Upregulated proteins included those associated with cellular stress response (e.g., DNA damage‐inducible 1 homolog 2) and immune activation, whereas proteins involved in hemoglobin clearance and mitochondrial metabolism were downregulated, consistent with hemolysis and mitochondrial dysfunction. These findings suggest that urinary proteomics can capture systemic processes relevant to SCD pathophysiology. Despite the small and heterogeneous cohort, this study highlights candidate urinary biomarkers and molecular pathways that warrant validation in larger, stratified cohorts to assess their potential for disease monitoring.

## 1. Introduction

Sickle cell disease (SCD) has a complex pathophysiology driven by hemoglobin S (HbS) polymerization, leading to erythrocyte sickling, vaso‐occlusion, and hemolysis. Free hemoglobin and heme released during hemolysis drive oxidative stress, endothelial dysfunction, and immune activation, thereby contributing to progressive organ damage [1–3]. Due to the complex and interconnected nature of these processes, reliable biomarkers are needed to better understand the underlying mechanisms involved in SCD and to explain its clinical heterogeneity.

Urine proteomics represents a promising noninvasive approach for biomarker discovery, as it reflects both systemic and kidney‐specific pathophysiological processes [4]. Mass spectrometry–based proteomics enables comprehensive urinary protein profiling and may reveal molecular signatures associated with disease progression and organ involvement in SCD.

This pilot study characterized the urinary proteome of individuals with SCD compared to healthy controls, aiming to generate hypotheses on disease‐related pathways and identify potential biomarkers for future research.

## 2. Materials and Methods

The study analyzed spot urine samples from pediatric and adult patients with SCD who are enrolled in the Radboud Biobank. The diagnosis of SCD was genotypically confirmed as HbSS, HbSβ^0^, HbSβ^+^, HbSC, or HbSD. Patients on different treatment regimens were included. Samples were collected between April 2018 and December 2023.

This study was conducted using data and biological samples from the Radboud Biobank as part of the SCORE consortium. Written informed consent was obtained from all participants or their legal guardians before inclusion in the Radboud Biobank. The study was conducted in accordance with the principles of the Declaration of Helsinki.

Mid‐stream urine samples were collected during routine clinical visits, processed within 4 h and stored at −80°C at the Radboud Biobank until analysis (Supporting Information 1). Control urine samples were refrigerated and processed within 24 h of collection. To minimize technical variation, all samples were processed in a single batch and aliquoted to avoid freeze‐thawing cycles.

For each measurement, a freshly thawed aliquot was used. After determining total urinary protein concentration, 10 μg of total protein from each sample was subjected to lysis, reduction, alkylation, and trypsin digestion using the PreOmics iST kit. Protein abundances, therefore, reflect relative abundances within an equal total protein input, rather than urinary protein concentration or creatinine‐normalized excretion. LC‐MS/MS analysis was performed on an Evosep One nanoflow liquid chromatography, connected to a timsTOF Pro2 instrument (Bruker Daltonics).

Protein identification and quantification were performed using data‐dependent acquisition (dda‐PASEF) and data‐independent acquisition (dia‐PASEF) workflows. Label‐free quantification was performed using match between runs (LFQ‐MBR) in PaSER (Supporting Information 1).

Proteomics data were filtered to retain proteins quantified in ≥ 75% of any group. Multivariate analysis, including principal component analysis (PCA), partial least squares–discriminant analysis (PLS‐DA), and genetic algorithm–random forest (GA‐RF), was applied to identify differentially expressed proteins between SCD patients and healthy individuals (Supporting Information 2). Proteins with positive or negative log_2_ fold change (FC) ≥ 0.58 (adjusted *p* value < 0.05) were selected for functional clustering using STRING (EMBL) to assess biological interactions [5].

## 3. Results and Discussion

In total, 12 pediatric and 20 adult patients with SCD (genotypes HbSS, HbSβ^0^, HbSC, and HbSD) in a steady‐state condition were included, with a median age of 20 years (range: 3–58 years), alongside 19 healthy controls with a median age of 40 years (range: 6–68 years). Chronic treatment regimens included hydroxyurea (*n* = 22), exchange transfusions alone (*n* = 2), and no treatment (*n* = 8) (Table 1).

**TABLE 1:** Demographic and laboratory characteristics of individuals with sickle cell disease.

	Total population
*Demographics*	
Age; median (range)	20.4 (3.1–58.3)

	**Number (%)**

Gender, female	17 (53.1)
Genotype	
‐ HbSS	25 (78.1)
‐ HbSβ^0^	1 (3.1)
‐ HbSC	5 (15.6)
‐ HbSD	1 (3.1)
Treatment	
‐ None	8 (25.0)
‐ Hydroxyurea (HU)	20 (62.5)
‐ Exchange transfusion (RCT)	2 (6.2)
‐ HU + RCT	1 (3.1)
‐ HU + erythropoiesis stimulating agent	1 (3.1)

**Laboratory results**	**Median (IQR)**

Hemoglobin (mmol/L)	6.3 (1.6)
MCV (fL)	85 (28)
HbF (%)	13.1 (16.6)
HbS (%)	61.9 (14.1)
LDH (U/L)	340 (142)
Serum creatinine (μmol/L)	51.4 (31.7)
GFR (mL/min/1.73 m^2^)	149 (35)
Albumin‐to‐creatinine ratio (mg/mmol)	0.99 (2.02)

*Note:* Table shows demographic and laboratory characteristics of individuals with sickle cell disease. HbF, fetal hemoglobin; HbS, sickle hemoglobin; LDH, lactate dehydrogenase; eGFR, estimated glomerular filtration rate (using the CKD‐EPI equation).

Abbreviation: MCV, mean corpuscular volume.

### 3.1. Urinary Proteome Differences Between SCD and Healthy Controls

LC‐MS/MS analysis identified 2263 distinct urinary proteins, with 978 consistently detected in ≥ 75% of samples from any group. Unsupervised PCA score plots and PLS‐DA revealed clear separation between SCD patients and healthy controls (Figure 1).

FIGURE 1Different representations of differentially expressed proteins. (A) Principal component analysis (PCA) score plot of 90 differentially expressed urinary proteins identified by PLS‐DA (VIP‐score > 1, *q* < 0.05), showing significant separation between SCD patients and healthy controls along PC1 (32.7% of total explained variance). (B) The scree plot confirms that the majority of variance in the data is explained by PC1. (C) Heatmap of hierarchical clustering analysis. Red represents SCD patients, while blue denotes healthy controls. Greyscale tones indicate protein abundance, with darker shades representing higher protein abundance. PLS‐DA: partial least squares–discriminant analysis, PC: principal component.

**Figure:**
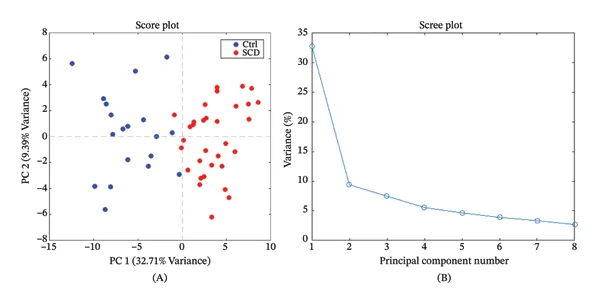

**Figure:**
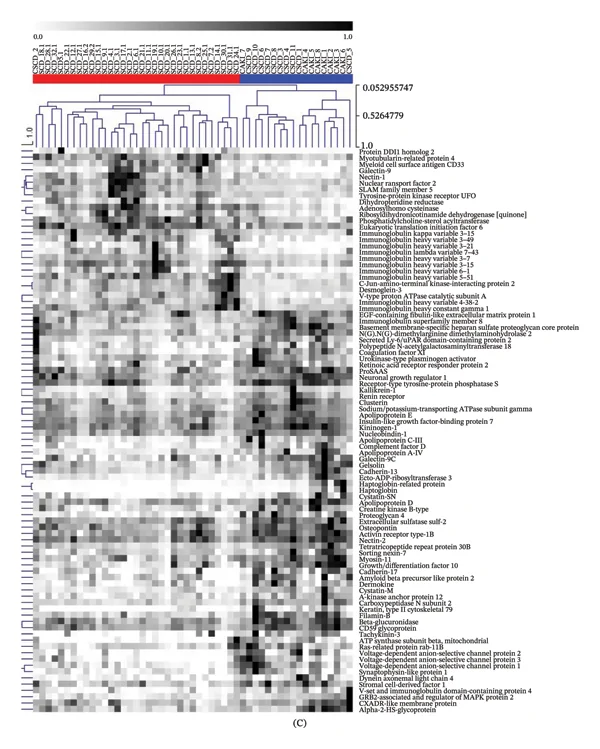

Ninety proteins were differentially expressed between SCD patients and healthy controls, with 26 upregulated and 64 downregulated in the urinary proteome of SCD patients (Supporting Table 1). PLS‐DA did not yield valid models for differentiating urinary proteome by gender, hydroxyurea treatment, clinical subphenotypes, and genotypes, suggesting limited effects of these factors on urinary proteome composition in this small cohort (data not shown).

### 3.2. Upregulated Proteins: Cellular Stress Response and Immune Activation

#### 3.2.1. DDI2: A Marker of Proteasome Adaptation and Ferroptosis

In our SCD cohort, DNA damage‐inducible 1 homolog 2 (DDI2) was the most strongly upregulated urinary protein (log_2_ FC: 6.2). DDI2 is an aspartyl protease that senses proteasome overload—marked by the accumulation of ubiquitin‐tagged proteins—by cleaving nuclear factor erythroid 2–related factor 1 (NRF1). Once cleaved, NRF1 translocates to the nucleus to induce expression of proteasome genes, thereby restoring protein degradation and protecting cells from proteotoxic stress [6–9]. This compensatory mechanism parallels observations in multiple myeloma, where DDI2‐NRF1 activation contributes to drug resistance under prolonged proteasome inhibition [10].

Evidence from SCD mouse models and patients shows that hemoglobin oxidation induces ubiquitin overload and proteasome stress, effects that are partially relieved by hydroxyurea [11, 12]. In our cohort, most patients were on hydroxyurea; however, urinary DDI2 abundance did not differ significantly between hydroxyurea‐treated and hydroxyurea‐untreated groups, suggesting persistent proteasome stress despite therapy.

Beyond proteasome regulation, DDI2‐NRF1 signaling has also been linked to ferroptosis, an iron‐dependent, nonapoptotic form of cell death triggered by oxidative stress [9]. In SCD, iron overload from chronic hemolysis and transfusions drives reactive oxygen species (ROS)–driven lipid peroxidation and ferroptotic pathways, which may contribute to complications such as renal medullary carcinoma—where ferroptosis drives oncogenic signaling—and impaired wound healing in chronic leg ulcers due to the cutaneous iron accumulation [9, 13, 14].

Thus, increased urinary DDI2 in SCD patients likely reflects adaptation to ongoing oxidative and proteasome stress and may also signal ferroptotic injury. These findings highlight the interplay of proteasome regulation, oxidative stress, and ferroptosis in SCD, supporting DDI2 as a candidate urinary biomarker.

#### 3.2.2. Immunoglobulins and Immune Activation

Several immunoglobulin variants were found to be upregulated in the urine of SCD patients, particularly immunoglobulin heavy variables (IGHVs; log_2_ FC ranging from 0.96 to 3.44), suggesting enhanced immune activity. Remarkably, immunoglobulin heavy constant gamma 1 (IgG1; log_2_ FC: 0.98) was significantly increased despite its large molecular size (150 kDa) and hydrodynamic radius (62 Å), which typically limits its filtration through the glomerular barrier.

Increased urinary IgG excretion can occur in the context of altered glomerular hemodynamics, even in the absence of albuminuria [15, 16]. In SCD, glomerular hyperfiltration is a well‐recognized early manifestation of renal involvement [3, 17]. The absence of overt albuminuria in this cohort, therefore, suggests that the presence of urinary IgG1 may reflect early functional changes such as hyperfiltration, rather than overt structural glomerular damage.

These findings raise the possibility that urinary IgG1 could serve as a noninvasive marker for early renal vascular alterations in SCD, potentially enabling detection of subclinical kidney dysfunction before the onset of more overt clinical signs.

### 3.3. Downregulated Proteins: Hemolysis and Mitochondrial Dysfunction

The most strongly downregulated proteins were hemoglobin‐binding proteins, including haptoglobin and haptoglobin‐related protein (log_2_ FC: −5.7 and −4.4), consistent with ongoing hemolysis in SCD [1]. Concurrently, several proteins involved in mitochondrial metabolism were also reduced, particularly voltage‐dependent anion channel proteins (VDAC 1–3) and ATP synthase subunit β (ATP5B, or complex V). This pattern suggests a complex interaction between hemolysis and mitochondrial dysfunction in SCD.

Mitochondrial abnormalities are increasingly recognized in SCD pathophysiology. In particular, impaired mitophagy leads to the retention of mitochondria in mature erythrocytes, which normally lack these organelles [18]. VDAC1, located in the outer mitochondrial membrane, has been reported to play a crucial role in regulating mitochondrial permeability and mitophagy; its downregulation has been associated with mitochondrial retention [19]. Persisting mitochondria in erythrocytes increase oxygen consumption and the generation of ROS, exacerbating oxidative stress [18]. ATP5B is essential for mitochondrial energy production and respiratory function. Its dysfunction can impair mitochondrial respiration, increase mitochondrial membrane potential, and promote excessive production of ROS in sickled platelets, thereby contributing to platelet activation [20, 21].

Together, the reduced abundance of these mitochondrial proteins, along with the lower abundance of hemoglobin‐binding proteins in urine may reflect a cascade where hemolysis‐induced mitochondrial dysfunction amplifies oxidative stress and cellular injury, further driving the sickling process and disease progression.

Given the paucity of urinary proteomic studies in SCD, limited overlap with published data should be interpreted in light of the differing biological matrices and study designs, while also highlighting the complementary value of the present urinary dataset [22, 23].

This exploratory pilot study has several limitations. The small, heterogeneous cohort—including both pediatric and adult patients on different treatments—may introduce variability, as renal physiology and disease progression differ with age and therapy [24]. Although subgroup and post hoc analyses did not reveal significant differences based on age or treatment, these factors may still affect the urinary proteome. The absence of gender‐, race‐, and age‐matched controls may further affect comparability, given known demographic influences on proteomic profiles [25]. Additionally, without paired serum samples, we could not determine whether the observed urinary changes reflect systemic or kidney‐specific processes. The lack of overt signs of kidney damage may suggest a systemic contribution, although this cannot be concluded with certainty.

Finally, this retrospective study relied on spot urine samples collected during routine clinical visits, rather than standardized second‐morning voids [4]. Variations in sampling time, despite standardized collection and storage protocols, may have introduced inconsistencies.

## 4. Conclusion

This pilot study provides a comprehensive analysis of the urinary proteome in individuals with SCD, identifying differentially expressed proteins associated with proteasome regulation, immune activation, and mitochondrial dysfunction. The marked upregulation of DDI2 suggests an adaptive cellular response to stress, potentially aimed at compensating for oxidative damage. Conversely, the downregulation of hemoglobin‐binding proteins and mitochondrial proteins may indicate protective cellular mechanisms against hemolysis‐induced oxidative injury.

Together, these findings provide a first step toward noninvasive biomarkers for disease monitoring in SCD. Validation of these candidate biomarkers using targeted proteomic approaches in larger, age‐ and race‐matched cohorts, preferably with longitudinal follow‐up data, is warranted to assess their clinical relevance.

## Author Contributions

Saskia E. M. Schols designed the study. Jolein Gloerich contributed to the experimental design and data generation. Hans Wessels performed data analysis, and Vera Hoving conducted additional data analysis and wrote the initial draft. Saskia E. M. Schols, Albertine E. Donker, Dorine W. Swinkels, Miranda van Berkel, Hans Wessels, and Jolein Gloerich reviewed the manuscript. Vera Hoving wrote the final version of the paper.

We confirm that ChatGPT was not used in the preparation of this manuscript.

## Funding

No funding was received for this manuscript.

## Ethics Statement

Written informed consent was obtained from all participants or their legal guardians at inclusion in the Radboud Biobank, allowing storage of samples for future research. The study was conducted under the Radboud Biobank’s provisions, approved by the Medical Research Ethics Committee (MREC) Oost‐Nederland (protocol number 2016–2362, 11 May 2016). Healthy controls provided written consent for a single urine collection, a procedure considered non‐WMO and not requiring ethics review. Data were processed in accordance with GDPR and institutional privacy standards, and the study adhered to the Declaration of Helsinki.

## Conflicts of Interest

The authors declare no conflicts of interest.

## Supporting Information

Additional supporting information can be found online in the Supporting Information section.

## Supporting information


**Supporting Information** Supporting Information 1. Detailed materials and methods describing patient/sample selection, urine sample collection and preparation, LC‐MS/MS proteomic analysis, and statistical and bioinformatic data analysis. Supporting Information 2. Detailed PLS‐DA model performance for distinguishing patients with sickle cell disease from healthy controls, including model validation and performance parameters. Supporting Table 1. Differentially expressed proteins in the urinary proteome of SCD patients compared with healthy controls. The table presents the average protein expression in the SCD and healthy control groups, together with the log_2_ fold change (SCD/HC). A positive log_2_ fold change indicates higher protein abundance in SCD patients compared with healthy controls, whereas a negative log_2_ fold change indicates lower protein abundance. Abbreviations: SCD, sickle cell disease; HC, healthy controls; log_2_ FC, log_2_ fold change.

## Data Availability

Data are available from the corresponding author upon reasonable request.
