# Supplementary material for: Urinary Proteomic Signatures in Sickle Cell Disease: Search for Biomarkers Associated With Underlying Molecular Pathways
Source: Adv Hematol. 2026 Sep 15;2026:8684436. doi: 10.1155/ah/8684436 (PMC13578929; doi:10.1155/ah/8684436)
Supplement: Supplementary file 1 [file AH-2026-8684436-s001.docx]

**Supplemental Information 1.**

Materials and methods
*Sample selection*
This study analyzed spot urine samples from pediatric and adult patients with sickle cell disease who are enrolled in the Radboud Biobank. Diagnosis was genotypically confirmed as HbSS, HbSβ^0^, HbSβ^+^, HbSC or HbSD. Patients on different treatment regimens were included. Samples were collected between April 2018 and December 2023.

Patients with homozygous HbSS or compound heterozygous Hbβ^0^ were further categorized based on their predominant subclinical phenotype:
- Hemolysis-dominant phenotype: Hemoglobin <10.4 g/dL with reticulocytosis and elevated LDH, before treatment initiation.
- Vaso-occlusive dominant phenotype: Hemoglobin >10.4g/dL, independent of hemolytic parameters.
Patients with HBSC or HbSD were grouped together.

Patients were recruited during routine outpatient visits. Written informed consent was obtained from all participants or their legal guardians before inclusion at the Radboud Biobank. The study complied with the Declaration of Helsinki and was approved by the Radboudumc Institutional Review Board (protocol number: 2013-064).

*Sample collection*
Spot urine samples were collected concurrently with routine diagnostic tests.
Mid-stream urine samples were obtained during routine clinical diagnostic care. After collection, urine was centrifuged at 2,000 x *g* for 10 minutes at 4˚ C and urine samples were frozen at -80˚ C within 4 hours after collection in 9mL aliquots.

*Protocol sample preparation*
After centrifugation of the urine samples at 10,000 x *g* for 5 minutes. Total urinary protein quantification was performed using a 2D Quant Kit (GE Healthcare) according to the manufacturers’ instructions.

Samples were filtered, centrifuged at 14,000 x *g* for 80 minutes, mixed, concentrated to 50ug, and centrifuged for an additional 30 minutes.

Equal amounts of total protein were subjected to lysis, reduction, alkylation and trypsin digestion according using the PreOmics iST kit (PreOmics), and the manufracturers’ protocol for urine samples was used.

*LC-MS/MS*
All liquid chromatography-tandem mass spectrometry (LC-MS/MS) measurements were performed using an Evosep One nanoflow liquid chromatograph (Evosep) connected to a timsTOF Pro2 instrument (Bruker Daltonics) via a CaptiveSprayer nanoflow electrospray ionization source, with real-time data processing by PaSER (Bruker Daltonics). For all measurements, the equivalent of 10ug total protein was used as input.
First, a spectral library was constructed by measuring each sample using the pre-defined 30SPD LC method using a 0.150mm ID x 150mm length C18 reversed phase column (EV1137 performance column) in combination with the default data-dependent acquisition Parallel Accumulation Serial Fragmentation (dda-PASEF) method for long LC gradients. Acquired fragmentation spectra were searched in real time against canonical human protein sequences (UniprotKB) using the ProLuCID workflow (PaSER v2023) with following parameters: 20ppm precursor and fragment ions tolerance, tryptic specificity, maximum of 1 missed cleavages, fixed carbamidomethylation of Cys, variable oxidation of Met, maximum FDR tolerance set to 1% at protein, peptide and peptide spectrum match levels (1). Spectra that passed the FDR filtering were used to construct a human urinary spectral library for subsequent data independent acquisition experiments.
Next, each sample was measured again using the default data-independent acquisition PASEF (dia-PASEF) method for long LC gradients to generate the urine proteome dataset for subsequent biomarker discovery. Spectra were searched against the constructed human urinary spectral library using the TIMS DIA-NN workflow with the following parameters: DIA scan mass tolerance: 20ppm, MS1 mass tolerance: 15ppm, Q-value filters: 0.1, match-between-runs retention time tolerance: 1minute.(2)

*Data analysis*
Proteomics data were first filtered to select proteins quantified in at least 75% of the samples in any sample group for further analysis. Univariate and multivariate data analysis were performed in MATLAB software (v. R2014b;(3)) using previously published scripts for PCA and PLS-DA.(3)
Briefly, PCA was performed on autoscaled LFQ value. The PLS-DA model was constructed using a repeated double cross-validation procedure with 21 model iterations. The number of latent variables was optimized in a Leave-One-Out inner cross-validation loop, and classification models were built using a 3-fold outer cross-validation loop. PLS-DA features with variable importance in project (VIP) > 1 and with false discovery rate (FDR) < 5% (based on 2000 permutations) were selected as discriminant features from the model.
For results interpretation, proteins with a positive or negative log_2_ fold change ≥0.58 were selected for functional clustering using STRING (EMBL) to assess biological interactions.(4)

Categorical data were summarized as frequencies and percentages, while continuous variables were presented as median and interquartile range (IQR), unless specified otherwise. Mann-Whitney U-test or Kruskal-Wallis tests were applied to the subset of proteins selected by PLS-DA to assess significant differences in their expression between clinical subphenotypes, hydroxyurea treatment groups (with or without treatment), and between age groups (children and adults).
All statistical analyses were conducted using IBM SPSS Statistics version 29.0 for Windows (Statistical Package for the Social Sciences).

**Supplemental Information 2. PLS-DA models**

**Valid PLS-DA model: SCD patients vs. healthy controls**
PLS-DA performance parameters:
AUC: 0.9072
Accuracy: 0.9104
Sensitivity: 0.8947
Specificity: 0.8817
Fscore: 0.9196

Settings: k-fold crossover: 3, 21 iterations, 2000 permutations

**Supplemental Table 1. Overview of Differentially Expressed Proteins in the Urinary Proteome Between SCD Patients and Healthy Controls**

| A. Increased expression | | | | |
| --- | --- | --- | --- | --- |
| ID | **Protein description** | **AVG SCD** | **AVG HC** | **log2 FC ratio** |
| Q5TDH0 | Protein DDI1 homolog 2 | 3881,30 | 51,54 | 6,23 |
| A0A0B4J1V1 | Immunoglobulin heavy variable 3-21 | 3857,57 | 355,34 | 3,44 |
| A0A0B4J1U7 | Immunoglobulin heavy variable 6-1 | 20054,47 | 3343,29 | 2,58 |
| O00182 | Galectin-9 | 5625,14 | 1149,00 | 2,29 |
| P20138 | Myeloid cell surface antigen CD33 | 11561,05 | 2488,05 | 2,22 |
| A0A0A0MS15 | Immunoglobulin heavy variable 3-49 | 6213,48 | 1505,23 | 2,05 |
| P09417 | Dihydropteridine reductase | 7102,60 | 1780,62 | 2,00 |
| P38606 | V-type proton ATPase catalytic subunit A | 9120,62 | 2391,17 | 1,93 |
| P16083 | Ribosyldihydronicotinamide dehydrogenase | 19133,90 | 5591,62 | 1,77 |
| P04211 | Immunoglobulin lambda variable 7-43 | 20744,90 | 6124,35 | 1,76 |
| A0A0C4DH38 | Immunoglobulin heavy variable 5-51 | 14571,36 | 4764,57 | 1,61 |
| Q15223 | Nectin-1 | 7850,05 | 2675,47 | 1,55 |
| P23526 | Adenosylhomocysteinase | 18885,89 | 7249,37 | 1,38 |
| Q13387 | C-Jun-amino-terminal kinase-interacting protein 2 | 29676,78 | 12598,98 | 1,24 |
| P61970 | Nuclear transport factor 2 | 372712,47 | 162758,84 | 1,20 |
| P32926 | Desmoglein-3 | 228230,65 | 100380,63 | 1,19 |
| P0DP08 | Immunoglobulin heavy variable 4-38-2 | 31233,28 | 14217,77 | 1,14 |
| Q9UIB8 | SLAM family member 5 | 74520,63 | 33991,33 | 1,13 |
| P30530 | Tyrosine-protein kinase receptor UFO | 208365,09 | 96305,67 | 1,11 |
| P04180 | Phosphatidylcholine-sterol acyltransferase | 19470,71 | 9156,36 | 1,09 |
| P01624 | Immunoglobulin kappa variable 3-15 | 184931,50 | 91360,27 | 1,02 |
| A0A0B4J1V0 | Immunoglobulin heavy variable 3-15 | 66249,33 | 33445,06 | 0,99 |
| P01857 | Immunoglobulin heavy constant gamma 1 | 4388750,00 | 2228768,58 | 0,98 |
| P01780 | Immunoglobulin heavy variable 3-7 | 96284,28 | 49612,62 | 0,96 |
| Q9NYA4 | Myotubularin-related protein 4 | 145681,60 | 82591,45 | 0,82 |
| P56537 | Eukaryotic translation initiation factor 6 | 34421,67 | 21237,11 | 0,70 |

| B. Decreased expression | | | | |
| --- | --- | --- | --- | --- |
| ID | **Protein description** | **AVG SCD** | **AVG HC** | **log2 FC ratio** |
| P00738 | Haptoglobin | 4266,11 | 220751,81 | -5,69 |
| P00739 | Haptoglobin-related protein | 25240,62 | 516356,35 | -4,35 |
| Q5XKE5 | Keratin, type II cytoskeletal 79 | 316,90 | 3881,94 | -3,61 |
| P45880 | Voltage-dependent anion-selective channel protein 2 | 380,82 | 2044,71 | -2,42 |
| P03951 | Coagulation factor XI | 1142,91 | 5276,68 | -2,21 |
| Q92954 | Proteoglycan 4 | 1224,10 | 5479,87 | -2,16 |
| O96015 | Dynein axonemal light chain 4 | 577,20 | 2582,13 | -2,16 |
| P06576 | ATP synthase subunit beta, mitochondrial | 418,17 | 1808,54 | -2,11 |
| P01037 | Cystatin-SN | 2817,52 | 11532,69 | -2,03 |
| Q9UHF0 | Tachykinin-3 | 538,11 | 2187,60 | -2,02 |
| P00746 | Complement factor D | 822,88 | 3329,83 | -2,02 |
| Q9Y279 | V-set and immunoglobulin domain-containing protein 4 | 1896,02 | 7623,06 | -2,01 |
| Q9Y277 | Voltage-dependent anion-selective channel protein 3 | 376,54 | 1494,04 | -1,99 |
| Q13508 | Ecto-ADP-ribosyltransferase 3 | 6449,68 | 25040,27 | -1,96 |
| Q12864 | Cadherin-17 | 576,21 | 2116,23 | -1,88 |
| P21796 | Voltage-dependent anion-selective channel protein 1 | 2021,75 | 6876,16 | -1,77 |
| Q16563 | Synaptophysin-like protein 1 | 405,07 | 1318,34 | -1,70 |
| P02656 | Apolipoprotein C-III | 3431,40 | 10764,49 | -1,65 |
| Q6P9A2 | Polypeptide N-acetylgalactosaminyltransferase 18 | 349,24 | 1073,44 | -1,62 |
| P06727 | Apolipoprotein A-IV | 36027,72 | 109994,92 | -1,61 |
| P55107 | Growth/differentiation factor 10 | 2353,05 | 7128,03 | -1,60 |
| O75369 | Filamin-B | 6991,67 | 20724,62 | -1,57 |
| Q15828 | Cystatin-M | 29923,72 | 85258,69 | -1,51 |
| Q02952 | A-kinase anchor protein 12 | 1141,01 | 3227,28 | -1,50 |
| Q6E0U4 | Dermokine | 2070,57 | 5845,23 | -1,50 |
| P22792 | Carboxypeptidase N subunit 2 | 31842,32 | 86997,01 | -1,45 |
| P98160 | Basement membrane-specific heparan sulfate proteoglycan core protein | 547112,46 | 1470512,84 | -1,43 |
| P06870 | Kallikrein-1 | 276251,64 | 707432,16 | -1,36 |
| O75787 | Renin receptor | 10759,67 | 26650,15 | -1,31 |
| Q06481 | Amyloid beta precursor like protein 2 | 1342,37 | 3309,25 | -1,30 |
| Q75VX8 | GRB2-associated and regulator of MAPK protein 2 | 20111,58 | 49441,96 | -1,30 |
| P48061 | Stromal cell-derived factor 1 | 2777,55 | 6514,72 | -1,23 |
| P12277 | Creatine kinase B-type | 3914,00 | 8757,02 | -1,16 |
| P0DP57 | Secreted Ly-6/uPAR domain-containing protein 2 | 34289,97 | 75076,51 | -1,13 |
| Q6DKI2 | Galectin-9C | 3224,10 | 7056,22 | -1,13 |
| Q8N4P2 | Tetratricopeptide repeat protein 30B | 27434,86 | 59618,13 | -1,12 |
| P06396 | Gelsolin | 165246,84 | 353283,86 | -1,10 |
| P55290 | Cadherin-13 | 142586,70 | 304638,12 | -1,10 |
| Q9UNH6 | Sorting nexin-7 | 1665,40 | 3534,20 | -1,09 |
| P35749 | Myosin-11 | 3710,45 | 7850,95 | -1,08 |
| P02765 | Alpha-2-HS-glycoprotein | 364575,10 | 763830,84 | -1,07 |
| Q99969 | Retinoic acid receptor responder protein 2 | 1719,94 | 3556,90 | -1,05 |
| Q15907 | Ras-related protein Rab-11B | 3583,17 | 6960,37 | -0,96 |
| O95865 | N(G),N(G)-dimethylarginine dimethylaminohydrolase 2 | 21645,25 | 41378,84 | -0,93 |
| P54710 | Sodium/potassium-transporting ATPase subunit gamma | 29061,20 | 54173,81 | -0,90 |
| Q969P0 | Immunoglobulin superfamily member 8 | 32555,87 | 59038,99 | -0,86 |
| Q9H6B4 | CXADR-like membrane protein | 7218,30 | 12648,80 | -0,81 |
| P08236 | Beta-glucuronidase | 22897,81 | 39801,85 | -0,80 |
| Q9UHG2 | ProSAAS | 27467,54 | 47054,73 | -0,78 |
| P36896 | Activin receptor type-1B | 28407,96 | 48513,88 | -0,77 |
| P10909 | Clusterin | 339912,70 | 576321,58 | -0,76 |
| Q12805 | EGF-containing fibulin-like extracellular matrix protein 1 | 27818,03 | 46589,51 | -0,74 |
| P13987 | CD59 glycoprotein | 922865,72 | 1538848,63 | -0,74 |
| P05090 | Apolipoprotein D | 2124990,53 | 3507894,74 | -0,72 |
| Q13332 | Receptor-type tyrosine-protein phosphatase S | 8964,89 | 14646,22 | -0,71 |
| P00749 | Urokinase-type plasminogen activator | 22531,07 | 35994,23 | -0,68 |
| P10451 | Osteopontin | 1979817,19 | 3102105,26 | -0,65 |
| Q02818 | Nucleobindin-1 | 16737,93 | 25680,62 | -0,62 |
| Q8IWU5 | Extracellular sulfatase Sulf-2 | 398805,88 | 602669,68 | -0,60 |
| P01042 | Kininogen-1 | 4184375,00 | 6171578,95 | -0,56 |
| Q7Z3B1 | Neuronal growth regulator 1 | 39849,05 | 58464,25 | -0,55 |
| P02649 | Apolipoprotein E | 50669,83 | 72691,37 | -0,52 |
| Q92692 | Nectin-2 | 91811,95 | 130917,07 | -0,51 |
| Q16270 | Insulin-like growth factor-binding protein 7 | 195825,45 | 277363,11 | -0,50 |

Overview of differentially expressed proteins in the urinary proteome between SCD patients and healthy controls. The values represent the average protein expression in the SCD group and healthy controls, followed by the log_2_ fold change of the SCD:HC ratio. A positive log_2_ FC indicates increased expression in SCD patients compared to healthy controls, while a negative log_2_ fold change reflects decreased expression.
*AVG: average value, SCD: sickle cell disease, HC: healthy controls, log_2_ FC: log_2_ fold change.*

**References**

1.	Xu T, Park SK, Venable JD, Wohlschlegel JA, Diedrich JK, Cociorva D, et al. ProLuCID: An improved SEQUEST-like algorithm with enhanced sensitivity and specificity. Journal of Proteomics. 2015;129:16-24.

2.	Demichev V, Szyrwiel L, Yu F, Teo GC, Rosenberger G, Niewienda A, et al. dia-PASEF data analysis using FragPipe and DIA-NN for deep proteomics of low sample amounts. Nature Communications. 2022;13(1):3944.

3.	Wessels HJCT, Kulkarni P, van Dael M, Suppers A, Willems E, Zijlstra F, et al. Plasma glycoproteomics delivers high-specificity disease biomarkers by detecting site-specific glycosylation abnormalities. Journal of Advanced Research. 2024;61:179-92.

4.	Szklarczyk D, Kirsch R, Koutrouli M, Nastou K, Mehryary F, Hachilif R, et al. The STRING database in 2023: protein–protein association networks and functional enrichment analyses for any sequenced genome of interest. Nucleic Acids Research. 2022;51(D1):D638-D46.
